# Visualizing human photoreceptor and retinal pigment epithelium cell mosaics in a single volume scan over an extended field of view with adaptive optics optical coherence tomography

**DOI:** 10.1364/BOE.393906

**Published:** 2020-07-22

**Authors:** Muhammad Faizan Shirazi, Elisabeth Brunner, Marie Laslandes, Andreas Pollreisz, Christoph K. Hitzenberger, Michael Pircher

**Affiliations:** 1 Center for Medical Physics and Biomedical Engineering, Medical University of Vienna, Waehringer Guertel 18-20, A-1090 Vienna, Austria; 2 ALPAO 727 rue Aristide Bergès 38330 Montbonnot-Saint-Martin, France; 3 Department of Ophthalmology and Optometry, Medical University of Vienna, Vienna, Waehringer Guertel 18-20, A-1090 Vienna, Austria

## Abstract

Using adaptive optics optical coherence tomography, human photoreceptors and retinal pigment epithelium (RPE) cells are typically visualized on a small field of view of ∼1° to 2°. In addition, volume averaging is required for visualizing the RPE cell mosaic. To increase the imaging area, we introduce a lens based spectral domain AO-OCT system that shows low aberrations within an extended imaging area of 4°×4° while maintaining a high (theoretical) transverse resolution (at >7 mm pupil diameter) in the order of 2 µm. A new concept for wavefront sensing is introduced that uses light mainly originating from the RPE layer and yields images of the RPE cell mosaic in a single volume acquisition. The capability of the instrument for in vivo imaging is demonstrated by visualizing various cell structures within the posterior retinal layers over an extended field of view.

## 1. Introduction

Adaptive optics optical coherence tomography (AO-OCT) [1–4] is a powerful imaging technique that enables the three dimensional visualization of individual retinal cells in vivo. With this imaging capability various different cell types such as cone photoreceptors [5–10], rod photoreceptors [11,12], blood constituents [13], retinal pigment epithelium (RPE) cells [14], Ganglion cells [15] and microglia [16] have been identified within the retina. In addition individual cells can be monitored over larger time periods enabling the visualization of physiological processes such as photoreceptor disc shedding [17].

However, the field of view (FoV) of these systems is typically limited to 1°∼2°, mainly because of aberrations varying across the FoV introduced by both, the imaging system and the eye. The angle where the aberrations can be assumed as constant is known as isoplanatic angle and varies between individuals [18]. For larger imaging beam diameters that are required for visualizing for example rod photoreceptors, this angle will be as small as 1°. Thus, typical systems record AO-OCT volumes with this limited FoV resulting in a difficult clinical translation of this technology as for many diseases larger patches of the retina need to be examined. Recording of a multitude of OCT volumes with this small FoV, followed by image stitching [19,20] is very time consuming and not feasible in patients. Another aspect is the determination of the imaged location that can be very difficult if image data is available only in a small FoV. For an extension of the FoV, the imaging beam diameter can be reduced [21,22], but this comes at the cost of lower transverse resolution.

Visualization of RPE and ganglion cells is of high clinical interest. Speckles that are inherently present in OCT imaging degrade the visibility of these cell types in single OCT scans. It has been shown that averaging of several OCT volumes reduces the appearance of speckles because of cell motility and allows for a clear visualization of these cell types [14,16,23]. However, recording of multiple OCT volumes that are spread over longer time periods is time consuming and sets high demands on a corresponding image registration software.

One key aspect for adaptive optics imaging is an accurate wavefront sensing. In AO-OCT this can be done with the implementation of a separate laser beacon [24] or by using part of the OCT imaging light that is diverted to a Shack Hartmann wavefront sensor via a pellicle beam splitter [25,26]. As wavefront sensing is not confocal, the light contribution from different layers can be problematic and may deteriorate the adaptive optics correction performance.

In this paper we introduce a spectral domain AO-OCT instrument that is capable to record high resolution images over an extended FoV. Aberrations introduced by the system over the scanning angle of 4°×4° are reduced by the implementation of lens based telescopes and careful system alignment. Wavefront sensing is performed using only the cross polarized component of the light that is backscattered from the retina that mainly originates from the RPE because of its depolarizing character. To stabilize wavefront sensing over the large field of view, the sensor exposure is synchronized with the slow scanner of the OCT system. Finally, we introduce a new post processing method for compensating B-scan rotation and retinal curvature that facilitates posterior retinal layer segmentation. With this instrument the cone and RPE cell mosaics are visualized over an extended FoV and with a single volume acquisition. Through image stitching of 7 volumes, a horizontal field of view of ∼22° is achieved. Imaging results are presented from healthy volunteers and the RPE cell density is quantified in six healthy subjects.

## 2. Methods

**Fig. 1.**
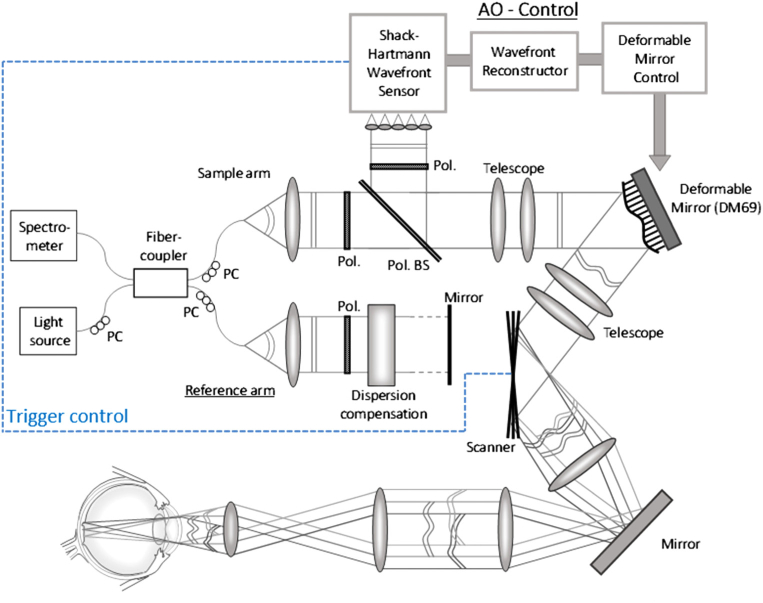
Scheme of the extended field of view adaptive optics optical coherence tomography setup. PC: polarization controller, Pol.: polarizer, Pol. BS: polarizing beam splitter. (For simplification only one galvanometer scanner is drawn and the corresponding telescope is omitted).

The system evolved from our previously presented adaptive optics scanning laser ophthalmoscope (AO-SLO) setup [27]. However, several modifications were necessary in order to allow for AO-OCT imaging. Thus, a scheme of the system is shown in Fig. 1. A light source (BLM-S-840, Superlum, Russia) with a center wavelength of 840 nm and a full-width-at-half-maximum of 50 nm is used, that yields a theoretical axial resolution of 4.5 µm (5 µm measured) in tissue. The single mode fiber output of the light source with 20 mW emission power is connected to a fiber coupler with a splitting ratio of 90:10 such that 10% of the light will be directed to the sample arm while 90% will be directed to the reference arm of the interferometer. Three polarization controller paddles are used at the source-, sample- and reference-arm fiber patches to adjust the polarization state of the light. In the sample arm, the light from the fiber output is collimated and traverses a Glan-Thompson polarizer (Pol.) with an extinction ratio of 100,000:1 in order to generate linear, horizontally polarized light. Then the beam traverses a polarizing cube beam-splitter (Pol. BS) that transmits the horizontally polarized light. The beam propagates through a first telescope (details of the lenses can be found in [27]) and is reflected by a deformable mirror (DM, Alpao 69, Grenoble, France).

The light passes through a second and a third telescope that image the plane of the DM onto the horizontal and vertical galvanometer scanners (GVS002, Thorlabs, Germany), respectively. Then the light passes through a fourth telescope that incorporates a flat mirror. In our previous setup a second deformable mirror was placed at this location for additional field correction in AO-SLO [27]. However, the used concept cannot directly be transferred to AO-OCT imaging. Finally, the light passes through the last telescope before it is incident onto the eye with a beam diameter of 7 mm. This yields a diffraction limited transverse resolution of ∼2.3 µm. An internal fixation target is presented to the subject by the use of a dichroic mirror before the last telescope. The light is backscattered by the retina and follows the same path back. At the polarizing beam splitter, only light in the same horizontal polarization state is coupled back into the single mode fiber and contributes to the OCT signal.

Light in an orthogonal polarization state is reflected by the polarizing beam splitter and traverses another Glan Thompson polarizer oriented orthogonal to the first one. The light is then directed to the custom developed Shack-Hartmann wavefront sensor [28] for measuring the wavefront aberrations. The use of the cross polarized light for wavefront sensing greatly attenuates the light that reaches the sensor from back-reflections at the lens surfaces that would otherwise distort the wavefront measurement. Thus, the lenses can be aligned without any tilt to the incident beam which minimizes aberrations introduced by these lenses. The power incident on the eye is 650 µW which is well below the limits for safe exposure according to the European light safety standards [29] and yielded a measured sensitivity of ∼86 dB. Apart from the power, the spectral shape of the light beam at the location of the eye is measured during system alignment as the spectral shape varies depending on birefringence introduced by the optical fibers. The two polarization paddles in the source and sample arm are then aligned in such a way that the obtained spectrum is identical to that directly measured from the light source. This procedure is necessary as not all spectral components of the light source are in the same linear polarization state and thus the spectral shape after passing through the polarizer in the sample arm can be deteriorated.

In the reference arm the light exiting the fiber is collimated and traverses a similar Glan-Thompson polarizer as in the sample arm for dispersion compensation and for matching the polarization state of the light in the sample arm. Additional glass components are placed in the reference arm to compensate for dispersion in the sample arm that is introduced by the lenses and the aqueous humor of the eye. The hardware dispersion compensation (using one glass material) was optimized in order to achieve an axial resolution in the model eye that is close to the theoretically expected one (without additional dispersion compensation in post-processing). The light is then back-reflected by a mirror that is mounted on a translation stage for adjusting the reference arm length.

The light is back coupled into the single mode fiber from both arms and is directed to a customized spectrometer that records the modulated spectrum caused by interference of the light returning from sample and reference arm, respectively. The spectrometer consists of a collimator, a diffraction grating, a focusing lens, and a line scan camera (spL4096-140 km, Basler, Germany). In order to increase the acquisition speed, the spectrometer is designed such that the full spectrum of the light is captured by using only the central 800 pixels of the camera. As a result, an imaging speed of 250 kHz A-scan rate is achieved. The measured imaging depth range of the system is 2.36 mm.

### 2.1 Wavefront sensing and AO correction

For wavefront sensing only light returning from the retina that is in an orthogonal polarization state compared to the incident polarization state is used. This configuration greatly attenuates reflexes originating from the lens surfaces or from the cornea of the imaged subject. In addition, mainly light backscattered from the retinal pigment epithelium will be measured as this layer is known to depolarize the light [30–32]. As a consequence, the AO correction will converge specifically to this thin layer as light components originating from other layers are greatly attenuated. The measured wavefront is then used to drive the DM that is conjugated to the pupil plane of the eye for AO correction. The exposure time of the Shack-Hartmann wavefront sensor depends on the backscattered light intensity and is typically 50 ms yielding a potential AO correction rate of 20 Hz. As a large FoV is scanned, the measured wavefront will slightly change depending on the scanning position. Scanning along a B-scan (x-direction) is fast compared to the exposure time of the sensor, which results in a measurement of an averaged wavefront over the FoV. In the slow scanning (y-) direction, the time needed for full y-scanning is longer than the exposure time of the sensor. This causes fluctuations in wavefront measurements (and degradation of AO performance) as the sensor is exposed at varying y-scanning positions. In order to stabilize the wavefront measurement and to improve the AO correction we synchronized the wavefront measurement with the OCT scanners by implementation of a hardware trigger that starts the camera exposure of the wavefront sensor at exactly the same OCT y-scanning position at the center of the FoV. This results in wavefront measurements that are averaged over the same part of the AO-OCT field of view (that is smaller than the entire field of view of the AO-OCT).

### 2.2 Subject selection and scanning protocol

All measurements were performed according to the tenets of the Declaration of Helsinki and after approval of the study by the local ethics committee. Informed consent was obtained from each subject prior to the measurement after explaining the nature and form of the measurements. Healthy volunteers were recruited with the selection criteria of negligible media opacities and the ability to fixate stable. In total, 6 subjects participated in the study with a mean age of 32.8 (age range between 26 and 51 years). No drugs were administered for dilating the pupil as in most subjects the diameter of the pupil under the low light conditions in the lab was larger than 5 mm. Subjects were asked to fixate on an internal fixation target that was optically set to infinity. As accommodation of subjects has not been prevented, some data sets showed varying focus settings (associated with varying visibility of cone photoreceptors) and were discarded.

The instrument supports an imaging beam diameter of 7 mm and the scanning is performed over a FoV of 4°×4°. In the alignment and AO convergence mode, the beam is scanned over the entire FoV with a sparse sampling of 50 B-scans in the y-direction. This enables wavefront measurement and correction with a loop frequency in the order of 2-3 Hz. The AO algorithm automatically adapted to the observed pupil size. After subject alignment the AO-loop was closed and convergence of the loop below the Marechal criterion for diffraction limit (root mean square (RMS) error of the residual aberrations below λ/14 = 60 nm) is typically achieved within few seconds. After convergence, a volume acquisition is started (during this time the AO correction is not updated). For data recording we tested several sampling densities with the aim of good visualization of the cone mosaic pattern while minimizing motion artifacts. We found that a configuration of 750×750 pixels (lateral spacing of 1.6 µm in both directions) provided the best imaging results. The recording time of a single volume was ∼2.5 seconds with a B-scan rate of ∼300 frames per second.

### 2.3 Data processing

The recorded spectral data has been processed in a similar way as in standard OCT processing. The laterally averaged spectra are subtracted from each A-line spectrum before re-scaling of the spectral data into k-space. For dispersion compensation (dispersion mismatch of the measured eye to the model eye), a second order phase term, depending on the k-number was added. In order to compensate for rotation of the B-scan image (introduced for example by a slightly decentral pupil illumination) and curvature of the retina we introduce a new concept. For rotation compensation, a linear phase term was added to the spectral data depending on the A-scan position. Lateral subject motion (lateral pupil displacement) during data recording will result in a change of rotation. However, we did not observe noticeable changes in the rotation within a volume scan unless the scan contained severe motion artefacts and had to be discarded anyway. Axial motion may induce a rotation of the B-scan as well, but this influence was minimized by the short recording time of each B-scan. Finally, the residual curvature of the retinal image (that was noticeable in the 4°×4° FoV) was compensated by adding a linear phase term to each A-scan that depended quadratically on the A-scan location. This procedure has the advantage of a sub-pixel correction of the shape and rotation.

The determination of the corresponding phase terms was done manually on the first B-scan (by observing the corresponding rotation and deformation of the image) and after setting of these parameters the entire data set was evaluated by means of the Fast Fourier transform (FFT) with the same settings. As the correction for the retinal shape is done prior to FFT, the data has been zero padded only once in order to double the number of pixels within an A-scan. Axial displacement between B-scans was determined after the data processing procedure mentioned above, by averaging of all A-scans laterally and by peak detection of the first strong signal along the A-scan starting from the choroid to the anterior layers that corresponds to the RPE. With this information axial motion between B-scans was compensated. In a final step lateral motion is corrected by using cross correlation between successive B-scans. The motion corrected data is then stored as 3D stack and image planes corresponding to various retinal layers are retrieved for display.

## 3. Results

**Fig. 2.**
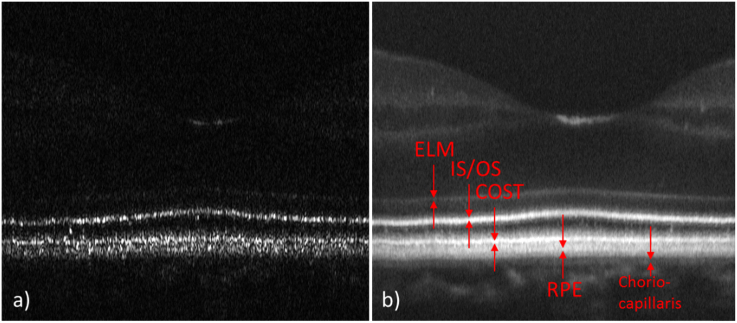
Representative AO-OCT B-scan images recorded at the fovea of a healthy volunteer. a) Single B-scan on a linear intensity scale, b) 50 adjacent B-scans averaged and displayed on a logarithmic intensity scale. ELM: external limiting membrane, IS/OS: junction between inner and outer segments of cone photoreceptors, COST: cone outer segment tips, RPE: retinal pigment epithelium. The lateral field of view is 3.8°.

A total of six subjects were recruited for imaging. Figure 2 shows representative images of the fovea of Volunteer 1 on a linear intensity scale recorded with a single volume scan. Figure 2(a) is a single B-scan image extracted from a volume scan showing the fovea centralis, where the packing density of cones is highest, in the center of the image. The retinal layers corresponding to the junction between inner and outer segments of photoreceptors (IS/OS) and the cone outer segment tips (COST) do not appear continuous but as bands consisting of discrete, highly reflective spots that can be associated with individual photoreceptors. For a better assessment of the individual layers and for a demonstration of the good axial resolution an averaged B-scan on a logarithmic scale is shown in Fig. 2(b). Various layers of the retina can be clearly visualized and are labeled in the image. Apart from the labeled layers, a very diffuse band can be observed directly anterior to the COST layer, probably originating form apical processes of the RPE cells.

Posterior to the RPE layer but anterior to the choriocapillaris, another layer can be identified similar to reports from other groups [16,33], that corresponds to the distal part of the RPE or Bruch’s membrane. Visualization 1 shows a fly-through movie of all recorded B-scans. In order to visualize anterior layers in this movie (logarithmic intensity scale), the contrast is set in such a way that the photoreceptor band appears slightly saturated.

**Fig. 3.**
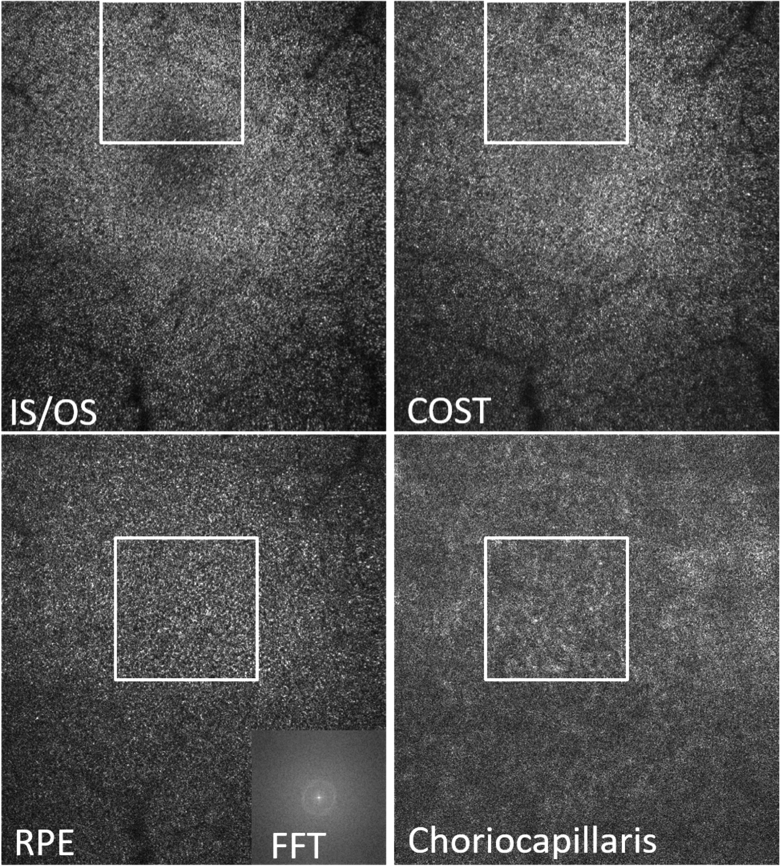
AO-OCT en-face images retrieved by depth integration of the layers shown in Fig. 2(b)). The white squares indicate region of interests that are presented as magnified views in Fig. 4. Field of view: 3.8° × 4°. IS/OS: junction between inner and outer segments of photoreceptors, COST: cone outer segment tips, RPE: retinal pigment epithelium, CC: choriocapillaris. FFT: 2D Fourier transform of the entire RPE image. In the lower row, the fovea centrals is located within the white square.

**Fig. 4.**
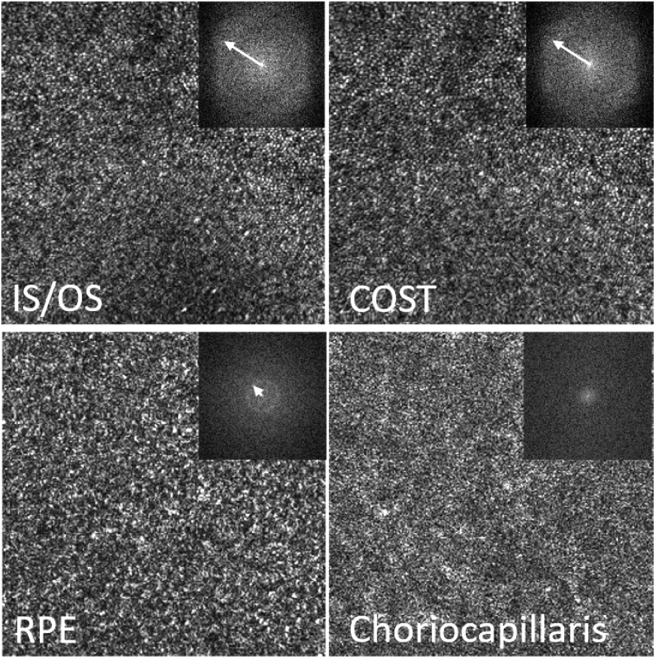
Magnified view of the regions of interest indicated with the white squares in Fig. 3. The insets show the corresponding FFT images of the regions of interest. The white arrow indicates the radius of Yellott’s ring. The dimension of the magnified image is 1.3° × 1.3°.

Corresponding en-face AO-OCT images that have been extracted at four different layers are displayed in Fig. 3 while Visualization 2 shows an en-face fly-trough (on a linear intensity scale) of the posterior retinal layers (IS/OS to choriocapillaris). After motion correction the obtained FoV is 3.8° (x) × 4° (y). In the periphery of Fig. 3(a) and (b) (displaying IS/OS and COST, respectively) the cone mosaic is clearly observable. As the FoV is quite large for photoreceptor imaging, the mosaic can be better seen in the enlarged regions of interest that are displayed in Fig. 4.

At the foveal center the visibility of the cone mosaic is less pronounced, specifically at the IS/OS layer as individual cones can only partially be resolved. At the level of the RPE, a regular cell mosaic can be seen (as additionally indicated by the 2D FFT of the entire image (cf. inset in Fig. 3)) mainly in the area of the foveal center while in the periphery it appears less prominent (cf. Figure 3 and Visualization 2). Similarly, at the level of the choriocapillaris, vessel like structures can be seen in the central part of the image while these structures are less visible in the periphery. In between the photoreceptor bands, within the outer segments, bright reflection spots are clearly visible (cf. Visualization 2) that probably originate from defects in the packing density of the cone outer segment discs [34].

Figure 4 shows a magnified view of the images corresponding to the white squares of Fig. 3. The FFT of the IS/OS and COST layers in this area show Yellott‘s rings [35] with the same radius, indicating a spatial frequency that corresponds to the photoreceptor density at this eccentricity. For the RPE cell mosaic, we can observe a ring with a much smaller radius indicating the regular spatial frequency of RPE cells that is smaller than that of photoreceptors. As the FFT shown in Fig. 4 is calculated only over the small area of this figure the ring is less pronounced than in Fig. 3.

**Fig. 5.**
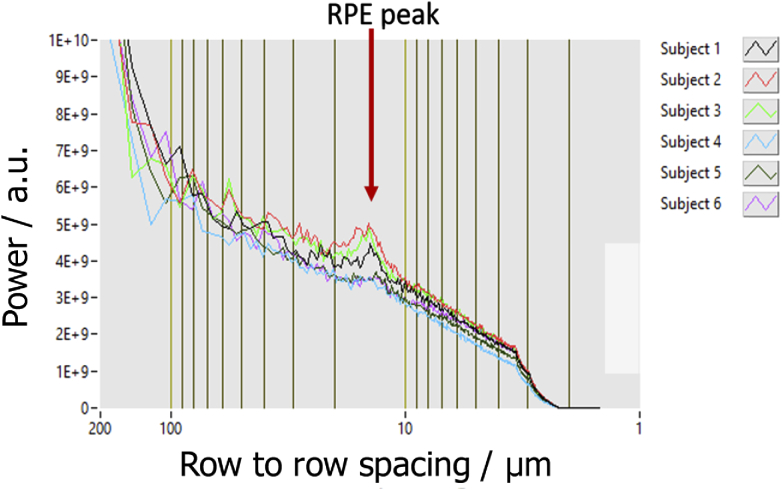
Spatial frequency of RPE cells that corresponds to the row-to-row spacing of these cells in six healthy subjects.

In order to quantify the spacing of RPE cells we performed the 2D FFT over the entire recorded image (as shown in Fig. 3) that produces a clearer ring. The FFT image was then converted to polar coordinates and the intensity was averaged for each radius value. The result of this procedure for images recorded in the fovea of six healthy subjects is shown in Fig. 5.

The determined mean row to row spacing between RPE cells was (13.9 ± 0.4) µm (range: 13.3 µm- 14.3 µm). These results are in good agreement with values known from histology and from previous reports [14,36]. The lower visibility of the ring in subjects 4-6 can be explained by the smaller pupil size during the measurements. The pupil sizes where retrieved from the Shack-Hartmann wavefront sensor images (7.75, 7.55, 6.9, 5.6, 5.6, 6.3 mm for subjects 1-6, respectively). In addition, the melanin concentration, an important factor for the speckle appearance of this layer, may be varying between subjects and may influence the RPE cell visibility as well.

**Fig. 6.**
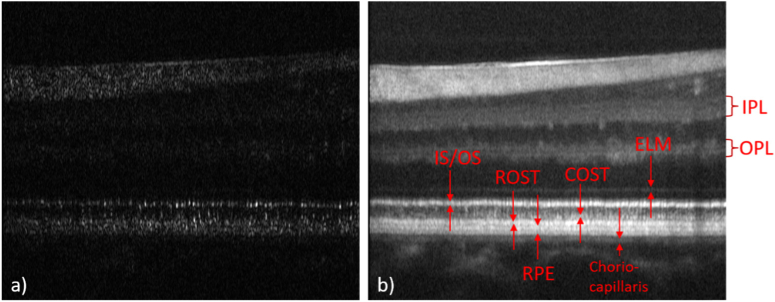
Representative AO-OCT B-scan images recorded at 5° eccentricity (nasal) to the fovea of a healthy volunteer. a) Single B-scan on a linear intensity scale, b) 50 adjacent B-scans averaged and displayed on a logarithmic intensity scale. ELM: external limiting membrane, IS/OS: junction between inner and outer segments of photoreceptors, COST: cone outer segment tips, ROST: rod outer segment tips, RPE: retinal pigment epithelium. IPL: inner plexiform layer, OPL: outer plexiform layer. The lateral field of view is 3.8°.

In a next step we recorded volumes at some eccentricity from the fovea. Figure 6 shows representative cross sectional image data recorded at 5 degrees eccentricity (nasal) from the fovea. Although the focus is set to the RPE layer, signal from anterior layers, specifically from the nerve fiber layer (topmost layer), can be seen in the single B-scan image (Fig. 6(a)) indicating a high sensitivity of the system. For this subject the pupil size was quite large (7.75 mm) which results in a small depth of focus and consequently in a blurring of the lateral structures in the anterior layers (cf. Visualization 3 that shows an en-face fly through movie of the 3D data set). As can be observed at the posterior retinal layers, the spacing between the cones at this eccentricity is much larger than in the fovea. In addition, the axial position of the cones appears to be more irregular specifically at the COST layer.

**Fig. 7.**
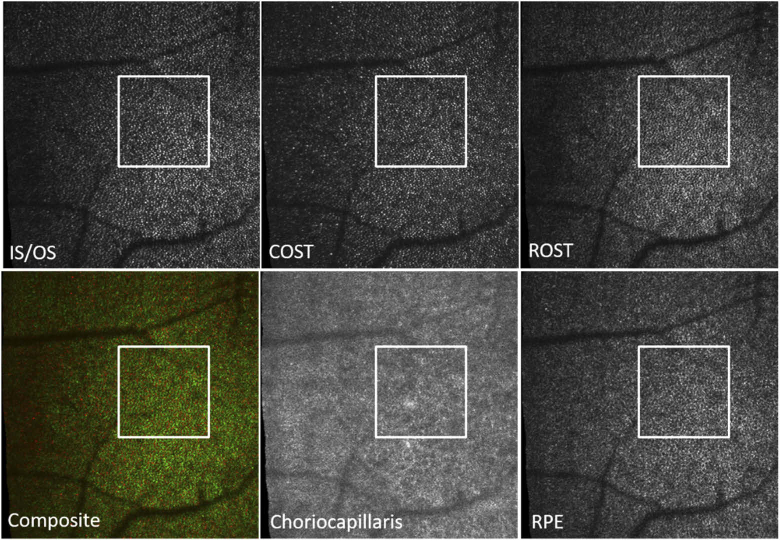
Representative AO-OCT en-face images recorded at 5 degrees eccentricity (nasal) from the fovea in a healthy subject. IS/OS: junction between inner and outer segments of photoreceptors, COST: cone outer segment tips, ROST: rod outer segment tips, Composite: false color image of COST (red) and ROST (green), RPE: retinal pigment epithelium, CC: choriocapillaris. Field of view: 3.8° × 4°.

The averaged B-scan displayed on a logarithmic scale (Fig. 6(b)) shows the individual layers more clearly. At this eccentricity the COST layer appears as diffuse and weakly reflecting layer mainly because the spacing of the cones is large and the signals are axially displaced with respect to each other. At this depth location, in the lateral space between the cones, no OCT signal can be observed (cf. Figure 7). In contrast to that, the IS/OS still appears as bright reflecting layer, as at this layer rods will contribute to the signal [11]. Posterior to the COST layer the rod outer segment tips (ROST) layer can be identified. Posterior to the RPE layer a second layer with similar reflectivity can be seen that may correspond to the distal part of the RPE cells or to Bruch’s membrane. Posterior to this band a weaker band corresponding to the choriocapillaris can be seen. In the anterior retina 3 distinct bands can be observed in the inner plexiform layer (IPL) and 2 distinct bands can be seen in the outer plexiform layer (OPL) indicating high axial resolution and good image registration.

**Fig. 8.**
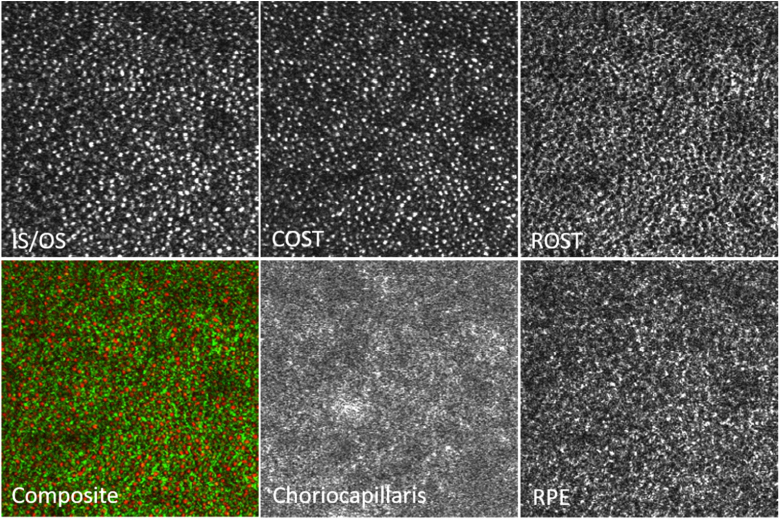
Magnified view of the regions of interest indicated with the white squares in Fig. 7. IS/OS: junction between inner and outer segments of photoreceptors, COST: cone outer segment tips, ROST: rod outer segment tips, Composite: false color image of COST (red) and ROST (green), RPE: retinal pigment epithelium, CC: choriocapillaris. The dimension of the magnified image is 1.3° × 1.3°.

Figure 7 shows the corresponding en-face images extracted from 5 layers that are indicated in Fig. 6. The full data set can be viewed in Visualization 3 as en-face fly through movie on a logarithmic scale. In the large FoV images, the observation of details is difficult as the total number of pixels of each image has to be reduced for display in this paper. Thus, we provide enlarged regions of interest (that are indicated by the white boxes in this figure) that can be viewed in Fig. 8. The cone photoreceptor mosaic can be clearly observed at IS/OS and COST layer. Posterior to the COST layer, the ROST layer, can be identified that shows hypo-reflective signals at locations of cones that are surrounded by hyper-reflective spots originating from the rod outer segment tips. In order to better visualize the correspondence of the cone locations in the ROST image we created a composite image where cones are color coded in red while the rods are color coded in green (cf. Figure 8) similar to previous reports [11,37]. Posterior to the ROST layer the RPE cell mosaic can be seen (cf. Figure 8) at a depth location of the anterior RPE band (cf. Figure 6(b)). Note the difference in the mosaic appearance between the ROST and RPE layers. Posterior to the RPE cell mosaic, another layer can be seen that does not show any regular structure and as pointed out, probably corresponds to the distal end of the RPE or Bruch‘s membrane. Posterior to this layer a faint layer corresponding to the choriocapillaris can be seen that shows in the large FoV image (cf. Figure 7) vessel like hyper-reflective patterns although no angiographic evaluation has been performed.

**Fig. 9.**
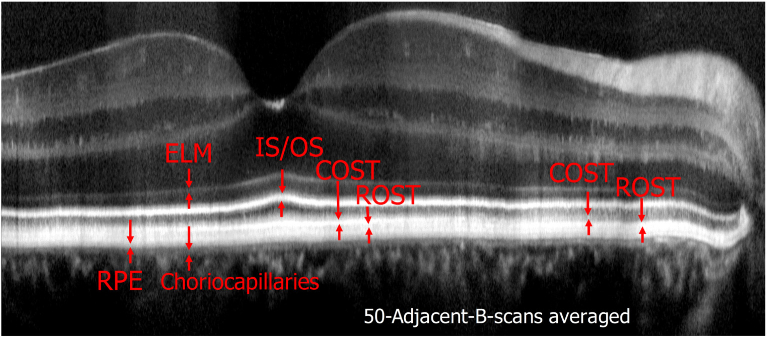
Averaged AO-OCT B-scan with an extended field of view generated by image stitching of 7 volumes. The lateral field of view corresponds to ∼ 22°.

In total we acquired retinal images of the same healthy volunteer at seven different locations spanning a horizontal FoV of ∼22°. The averaged B-scans were stitched together (using the Image J plugin “Mosaic J”) and can be viewed in Fig. 9. In this image the different retinal layers can be viewed over an extended FoV. The ELM and IS/OS are rather homogenous over the entire image. However, the COST layer, that is clearly visible at the fovea, slowly dissolves with eccentricity from the fovea in the temporal and nasal direction. On the other hand, starting at ∼2-3° eccentricity from the fovea towards the periphery, the ROST layer can be seen. In the fovea, posterior to the COST we observe a rather thick layer corresponding to the RPE that in fact can be split into two closely adjacent layers. These two layers become more distinct towards the optic disc. Posterior to this, a faint layer can be seen throughout the image that corresponds to the choriocapillaris.

**Fig. 10.**
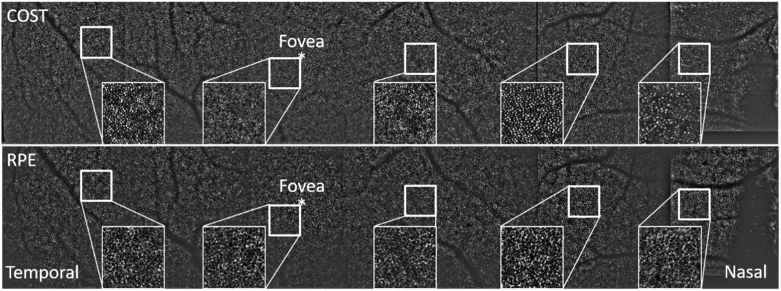
Stitched image (total of 7 volumes) of cone photoreceptor (top) and RPE (bottom) cell mosaics spanning a field of view of ∼22°. The orthogonal field of view is 4°. For a better visibility of the mosaics over this large field of view, a bandpass filter was applied. The insets are enlarged views of the region indicated with the white square.

In order to demonstrate the capability of the instrument for large FoV imaging, we present a stitched en-face view of the COST and RPE layers that were retrieved from the recorded volume data in Fig. 10. As the full pixel resolution of the large FoV image cannot be displayed in this paper format, we provide enlarged regions of interest as insets at various eccentricities from the fovea where both cell mosaics can be clearly seen. As the signal intensity varied between the recorded volumes, we applied the same band-pass filter (using Fiji image processing software with 1pixel-50pixels bandwidth) on all volumes (and image planes) that preserves the cellular structures (cones and RPE cells) but eliminates intensity fluctuations between volume scans. The missing information at the nasal side is due to segmentation errors at the papilla resulting from a deviation of the retinal curvature close to the optic disc. Thus, after our post processing steps the posterior layers in this area do not appear as horizontal lines as can be seen in Fig. 9.

## 4. Discussion

**Fig. 11.**
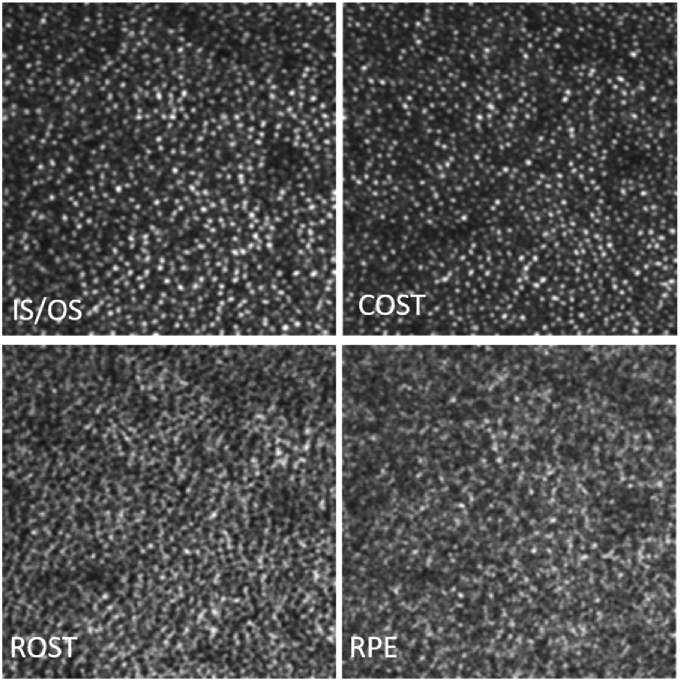
Magnified view of the regions of interest indicated with the white squares in Fig. 7 after application of a mean filter for a better visualization of cellular structures. Data was retrieved from a single volume scan. The dimension of the magnified image is 1.3° × 1.3°.

We presented imaging results with an AO-OCT instrument that is capable of recording images on a larger FoV while maintaining imaging with a large pupil size. Various cellular structures such as cone photoreceptors and RPE cell mosaics could be visualized with a single volume acquisition. The visibility of individual cells can be improved by applying a filter (using Fiji image processing software and applying a mean filter with radius of 1 pixel). The result is shown in Fig. 11 for the images displayed in Fig. 8. The large FoV enables an easier determination of the imaged region and a further extension by recording of multiple volumes and corresponding image stitching becomes feasible. Thus, retinal areas that are covered with standard OCT imaging can be imaged in reasonable time with this high resolution technology, simplifying quantitative assessments of cone photoreceptor or RPE cell densities or possibly classification of cone types [38] at various eccentricities from the fovea.

The RPE cell mosaic visualization capability of the instrument in a single volume acquisition is demonstrated by the visibility of Yellott’s ring in the FFT of the corresponding images (cf. Figures 3 and 4). This capability may be explained by the specific wavefront sensing technique that is implemented. As mainly light originating from the depolarizing RPE is used for wavefront sensing, all aberrations introduced to the imaging light returning from this layer will be minimized by the AO correction. In addition, the light backscattered from the RPE does not show the high directionality as light returning from the cones which might be beneficial for wavefront sensing. Thus, the highest transverse resolution and smallest (lateral) speckle size is obtained at the RPE layer and gives sufficient contrast to visualize the mosaic.

The importance of high lateral resolution for visualizing the RPE mosaic manifests itself by the lower visibility of the mosaic in those subjects whose pupil size was below 6.5 mm (our study was performed without artificially dilating the pupil). Here we want to point out that for visualizing cone photoreceptors (at some eccentricity from the fovea) minimizing of aberrations is not as critical as for the RPE, as cones show a high directionality of the backscattered light. This can be seen for example in Fig. 3 where the RPE mosaic can only be observed in the central part of the image (due to residual aberrations in the periphery) while the cone mosaic is still visible in the periphery. A clear benefit of a larger field of view is a better visibility of the ring assuming that the spatial frequency is constant over the entire field of view (cf. FFTs of the RPE that are shown in Figs. 3 and 4.) This enables the assessment of RPE cell densities even in the case of poorer mosaic visibility caused for example by a smaller pupil size as more RPE cells can be seen in the image that contribute to the generation of the ring.

Another aspect is a high axial resolution that is needed for separating the thin RPE layer showing the mosaic from other layers as pointed out in previous work [14]. We achieved that by careful alignment of the system to ensure that spectral components of the light source are not blocked, together with an optimized dispersion compensation in post processing. Finally, our method for correction of image rotation and of removing retinal curvature that is based on adding corresponding phase values to the spectral data prior to FFT enables an easy segmentation of the RPE layer. In contrast to other methods that use the evaluated images, sub-pixel axial displacements between A-scans can be achieved without the need of extensive zero padding which greatly reduces the amount of data in the processed volumes.

One current problem of our technique specifically for visualizing foveal cones is the rather low sampling density that is close to or even below the Nyquist criterion in the fovea centralis. In this work we used a compromise between high sampling density and short measurement times and found the used scanning pattern as best suited for visualizing structures outside the fovea. As subject motion remains an issue specifically for imaging patients with poorer fixation capabilities, recording of several volumes can be employed to increase the likeliness of obtaining a single volume with minimal motion artefacts.

Another issue in the fovea region are motion artifacts that are more pronounced as the imaging beam is visible to the subject and distracts from the fixation target. Both factors influence the en-face image quality in this region and thus the ability to clearly resolve foveal cones. However, the fovea is only a small part of the retina, and these cells could potentially be visualized on a smaller FoV by changing the scanning pattern. In addition, higher imaging speeds could be introduced that enable a higher sampling density and thus a better visualization of these small structures (at the cost of a lower overall sensitivity). This can be achieved by implementation of faster cameras, multiple cameras and corresponding switching schemes [26] or high speed swept sources [39].

Although the instrument had been designed to minimize aberrations introduced by the system itself, one remaining problem are aberrations introduced by the eye that vary across the FoV. This leads to a poorer image quality in some parts of the images (as can be observed in the corners of Fig. 3, on the left hand side in Fig. 7 or in the small missing parts of the mosaic of Fig. 10). We recently introduced a concept for SLO to overcome this problem [27]. The technique uses multi conjugated AO with two DMs to correct for these residual aberrations that vary across the FoV. In this work an image based metric is used to optimize the shape of the second DM (that corrects for aberrations varying across the field). However, a similar algorithm is not feasible for AO-OCT as the en-face image recording time is too long (2.5 seconds vs. 0.125seconds). Thus, a different concept is needed in order to translate this technology to AO-OCT.

The larger FoV in AO-OCT allows for exact determination of the imaged location and enables tracking of alterations in the retina occurring on a cellular level in a continuous fashion over a larger area instead of discretely sampling these structures in small separate imaging areas. For example, the RPE cell mosaic can be obtained over an almost uninterrupted area of 22° with a recording session of only 7 volumes (cf. Figure 10). This advantage gets enforced by the fact that we only need single volumes to retrieve such information, saving a lot of time on image acquisition and registration which is usually needed for averaging. This is enabled because of our improved wavefront sensing strategy which inherently optimizes the point spread function of the imaging beam at the RPE layer.

Another aspect of our system is the stabilization of the AO performance for the large FoV through the triggered sensor exposure. In the alignment mode only 50 B-scans are recorded while the exposure time of the wavefront sensor is 50 ms (corresponding to ∼15 B-scans). Thus, without triggering, the wavefront sensor would be exposed at varying locations along the slow scanning axis. Varying exposure locations usually cause fluctuations in the measured wavefronts and subsequently result in a degradation of AO correction. The possibility of generating B-scans that are of comparable size as obtained with commercial devices allows for a direct comparison to state of the art imaging devices and enables better interpretation of these images as the knowledge on the cellular composition of each layer as provided by AO-OCT is available. On the long term this may add highly relevant information for the clinical routine by simplifying the interpretation of images in a number of retinal diseases known to affect the RPE and photoreceptors such as age-related macular degeneration, central serous chorioretinopathy or inherited retinal dystrophies.

## 5. Conclusion

We presented an adaptive optics assisted spectral domain optical coherence tomography instrument that supports data acquisition on an extended FoV (4° × 4°). Various cell types such as cone photoreceptors, rods, RPE cells can be visualized in a single volume acquisition. The FoV can be further increased by stitching of several volumes, approaching the FoV of standard OCT imaging.
